# Seagrass ecosystems in peril: Climate change threatens blue carbon storage and ecosystem services

**DOI:** 10.1016/j.isci.2025.112909

**Published:** 2025-06-16

**Authors:** Linlin Song, Bohao He, Shahid Ahmad, Qian Li, Anping Chen, Wei Mao

**Affiliations:** 1 University of Chinese Academy of Sciences, Beijing 100049, China; 2 National Key Laboratory of Earth System Numerical Modeling and Application, Institute of Atmospheric Physics, Chinese Academy of Sciences, Beijing 100029, China; 3 Department of Electronics, Information, and Bioengineering, Polytechnic University of Milan, 20133 Milan, Italy; 4 Hainan Tropical Coastal Ecosystem Observation and Research Station, School of Ecology, Hainan University, Hainan 570228, China; 5 Department of Biology, Graduate Degree Program in Ecology, Colorado State University, Fort Collins, CO, USA

**Keywords:** Earth sciences, Marine geochemistry, Global change, Environmental management, Environmental monitoring, Aquatic science, Aquatic biology, Global carbon cycle

## Abstract

Climate change threatens seagrass ecosystems, which are vital for blue carbon sequestration and associated co-benefits. Our study on Hainan Island in the South China Sea assessed climate impacts on seagrass habitats using ensemble modeling under two scenarios (RCP4.5 and RCP8.5) for 2050 and 2100. We found the current seagrass carbon stock is approximately 1.194 Tg, but projections show habitat suitability could decrease by up to 74.78% by 2100 under the severe scenario, potentially causing economic losses of $1.02–1.27 billion. While seagrass restoration requires initial investment, it offers substantial long-term climate benefits. Our analysis indicates seagrasses may migrate toward higher latitudes due to climate change, though new habitats could emerge along Hainan’s southern coast by 2100. These findings emphasize the urgency of conservation and restoration efforts to safeguard seagrass ecosystems and their vital role in climate change mitigation strategies.

## Highlights

• Climate change threatens seagrass habitats and blue carbon storage potential
• Seagrass loss on Hainan Island may lead to significant blue carbon reduction
• By 2100, economic loss from seagrass decline estimated between $1.02 and $1.27 billion
• Seagrass restoration offers long-term benefits for coastal sustainability

## Graphical abstract

**Figure:**
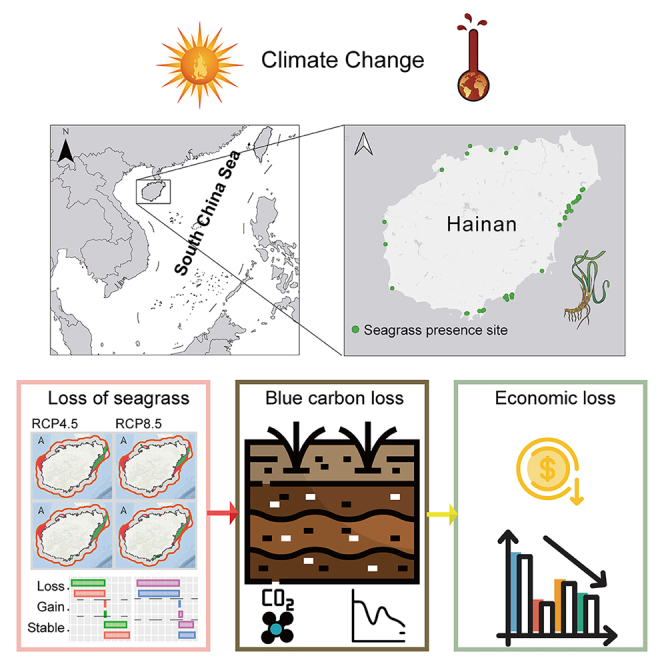

## Introduction

Biological invasions,^1^ climate change,^2^ and salinity fluctuations^3^ all pose significant threats to marine ecosystems, diminishing biodiversity, disrupting ecosystem functions, and undermining essential ecosystem services.^4^^,^^5^ The anticipated challenges are expected to alter local community structures through distribution shifts, new biotic interactions, and species extinctions.^6^ A crucial component of coastal ecosystems is the seagrass meadows, which consist of marine angiosperms that provide vital ecosystem services and support biodiversity.^7^ These meadows support many plants and animals, encompassing both endangered and commercially valuable species, inhabiting the vicinity of these meadows.^8^ Seagrass meadows offer a wide array of benefits, including fisheries production,^9^ water quality regulation,^10^ disaster resilience,^11^ and tourism,^12^ while also playing crucial roles in carbon sequestration nutrient cycling.^13^

The seagrass ecosystem spans all of the world’s five major bioregions, except for Antarctica.^14^ According to Fourqurean et al.,^15^ these meadows can accumulate up to 19.9 petagrams (Pg) of organic carbon. Despite covering less than 0.1% of the total ocean floor, they contribute approximately 10–18% of the oceanic carbon sink.^15^ Unlike terrestrial ecosystems, carbon stored in seagrass ecosystems, especially in sediments, remains sequestered for centuries to millennia.^16^ Seagrass meadows possess a greater carbon storage capacity than terrestrial forests. As the soil saturates with water, microbial carbon oxidation and release is deterred, leading to gradual carbon accumulation and thereby creating enduring carbon reservoirs.^17^ In marine sediments, seagrass meadows facilitate the long-term sequestration and preservation of carbon, making them crucial allies in climate change mitigation efforts.

Increasing pressures from climate change and anthropogenic activities have left seagrass habitats in peril,^18^^,^^19^ jeopardizing the continued provision of these essential ecosystem services. Global trends indicate a persistent decline in seagrass abundance, with more than half of the seagrass meadows disappearing since 2000.^15^^,^^20^ This decline is particularly alarming in the South China Sea, where seagrass ecosystems, despite their critical role in supporting global biodiversity, human welfare, and climate change adaptation and mitigation, remain poorly documented.^21^^,^^22^ While seagrass habitats’ distribution has been extensively studied in many regions,^23^^,^^24^ their blue carbon potential is infrequently mapped,^25^^,^^26^ especially in the South China Sea.

Over the last decade, there has been an exponential increase in global blue carbon research due to the promotion of climate change strategies under the Paris Agreement.^27^^,^^28^ The blue carbon strategy represents a key approach for combating and adapting to climate change by protecting and restoring coastal vegetation habitats. Many coastal countries around the world have adopted this strategy to evaluate blue carbon storage and net benefits of ecosystems.^29^ Therefore, assessing blue carbon at both global and regional scales is of paramount significance. However, the lack of data on seagrass distribution and blue carbon in the South China Sea region hampers the development of effective blue carbon protection and restoration strategies. Furthermore, it impedes reliable estimates of regional blue carbon storage and its overall ecological and economic value.

To address these knowledge gaps, understanding the potential shifts in seagrass distribution under future climate scenarios is crucial. Species Distribution Models (SDMs) provide a powerful methodological framework by using environmental data to correlate species occurrences with climatic conditions, enabling the prediction of species distributions under current and future scenarios.^30^ Effectively, SDMs delineate the functional niche of species by evaluating their relationships with environmental variables such as temperature, oxygen levels, salinity, pH, and other marine parameters.^31^^,^^32^ These models generate habitat suitability indices that can project how seagrass distributions might shift in response to changing ocean conditions, making them valuable tools for anticipating climate impacts on coastal blue carbon resources.^33^^,^^34^^,^^35^

To address these limitations, this study investigated how climate change will affect tropical seagrass ecosystems and their blue carbon potential on Hainan Island in the South China Sea through three interconnected hypotheses and corresponding research objectives. First, we hypothesized that climate change will significantly reduce seagrass habitat suitability on Hainan Island by 2100, with more extensive habitat loss occurring under the RCP8.5 scenario than under RCP4.5. To test this, we modeled projected range dynamics of seagrass species under different climate scenarios. Second, we hypothesized that this habitat reduction will cause substantial blue carbon losses with significant economic impacts that increase under more severe climate scenarios. Accordingly, we quantified potential carbon storage losses from seagrass die-offs and calculated their economic value, while also conducting spatial cost-benefit analyses to identify the economic costs and ecological benefits of restoring climate-threatened seagrass meadows. Third, we hypothesized that seagrass distributions will show distinct spatial migration patterns in response to climate change, with habitat centroids generally shifting latitudinally as ocean temperatures rise. To evaluate this, we analyzed the centroid position and migration distance of seagrass habitats under four distinct climate scenarios. Through this integrated approach, our study provides novel insights for the future research, protection, and management of tropical seagrass in the South China Sea region.

## Results

### Seagrass future habitat changes

**Figure 1:**
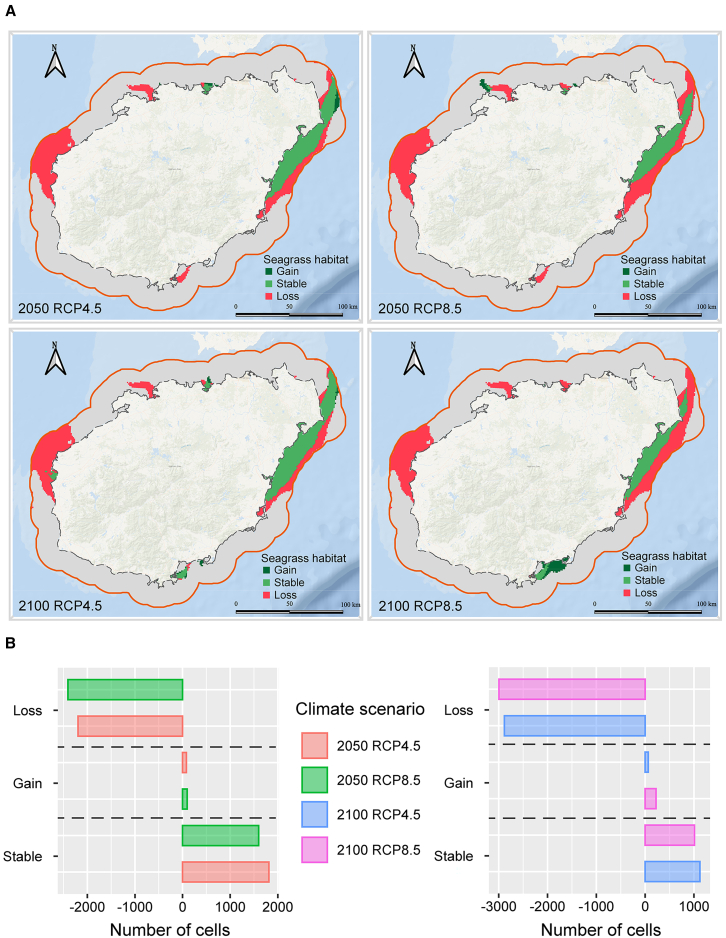
Predicted habitat changes under two climate scenarios (RCP4.5 and RCP8.5) for seagrasses in 2050 and 2100 (A) Map showing trends in the suitability of seagrass habitats under future climate scenarios. (B) Cell statistics of trends in the suitability of seagrass habitats under future climate scenarios.

As a result of climate change over time, seagrasses are indeed losing significant amounts of their blue carbon potential. Our model suggests that future climate change will generally lead to widespread carbon loss in seagrass habitats, However, a few habitats may experience an increase in carbon storage (Figure 1). Seagrass exhibits limited adaptability to climate change, and its ability to provide habitat is expected to diminish significantly, which may result in extensive losses. The eastern coast of Hainan Island is expected to remain suitable for seagrass habitats under all four climate scenarios in the future. Our findings suggest that under the low-emission scenario (RCP4.5), by 2050, seagrass may suffer losses of its suitable habitats, resulting in a blue carbon loss of 15404 Mg C ha^−1^. However, under the high-emission scenario (RCP8.5), by 2050, seagrass is predicted to lose 72% of its suitable habitats, leading to a blue carbon loss of 20248 Mg C ha^−1^. Moreover, the loss of suitable seagrass habitats is expected to intensify by 2100. Specifically, under RCP4.5, by 2100, seagrass could lose 60% of its suitable habitats, resulting in a blue carbon loss of 16904 Mg C ha^−1^. Whereas, under RCP8.5, by 2100, seagrass may lose 75% of its suitable habitats, leading to a blue carbon loss of 21029 Mg C ha^−1^. A climatic trajectory consistent with RCP8.5 is projected to result in significantly more carbon losses than RCP4.5 by 2050 and 2100. However, we also note that RCP8.5 in 2050 causes more carbon losses than RCP4.5 in 2100, suggesting that the worst-case global climate change scenario causes more severe losses than slower losses compounded over a longer period of time.

### Climate change leads to loss of seagrass carbon stocks

We estimated the total carbon stock of seagrass ecosystems on Hainan Island to be 1.194 Tg, with just under 4% stored in seagrass plant bodies and over 96% stored in sediment soils (Table 1). Compared to previously recorded data,^21^ the total carbon storage of Hainan Island is 29 times higher, indicating that tropical seagrasses on Hainan Island have high blue carbon potential. However, climate change will significantly decrease the range of seagrass habitats, resulting in the emission of carbon stored in the soil and exacerbating global warming. Our study found that under the RCP4.5 climate scenario, seagrass is expected to lose 0.63 Tg of carbon storage by 2050, while under the RCP8.5 climate scenario, it is expected to lose 0.84 Tg of carbon storage. By 2100, under the RCP4.5 climate scenario, seagrass is expected to lose 0.69 Tg of carbon storage, while under the RCP8.5 climate scenario, it is expected to lose 0.83 Tg of carbon storage. These results suggest that climate change will cause a significant decrease in seagrass carbon storage, and the rate of decline depends on the severity of the climate scenario; worse scenarios will result in greater losses of carbon storage.

**Table 1:** Seagrass blue carbon stock (plant and soil) estimates and seagrass blue carbon loss estimates under climate change scenarios

Scenario	Plant (Tg C)	Soil (Tg C)	Total (Tg C)	Loss (Tg C %)
Present	0.042	1.151	1.194	/
2050 RCP4.5	0.020	0.544	0.564	54.78
2050 RCP8.5	0.013	0.340	0.353	72.00
2100 RCP4.5	0.018	0.487	0.505	60.11
2100 RCP8.5	0.013	0.354	0.367	74.78

### Seagrass blue carbon economic analysis

Tropical seagrass on Hainan Island is projected to decline significantly under four future climate scenarios, leading to substantial losses of blue carbon (Table 2). By 2050, climate change is expected to cause a loss in blue carbon value from seagrass from $300 to $400 million, and this loss could potentially increase to between $1.02 and $1.27 billion if the conditions persist until 2100. Restoring the damaged seagrass meadow by 2050 would require an estimated investment of $160 to $220 million.

**Table 2:** The economic value of seagrass blue carbon lost due to climate change in 2050 and 2100 and the costs and benefits of its restoration

Scenario	Blue carbon economic loss (10^6^ US$)	Costs (10^6^ US$)	Benefits (10^6^ US$ year^−1^)
2050 RCP4.5	305.08	163.69	4.20
2050 RCP8.5	401.00	215.15	5.51
2100 RCP4.5	1018.74	179.62	4.60
2100 RCP8.5	1267.31	223.45	5.73

Despite this significant expense, restoring the seagrass meadow could generate $4.20–5.51 million per year in blue-carbon ecological benefits. On the other hand, restoring the seagrass until 2100 would entail a far more substantial cost of $1.02–1.26 billion than restoring it until 2050, but the associated blue carbon ecological gains are still relatively low at $4.60–5.73 million per year. The results suggest that the costs of restoring seagrass ecosystems may outweigh the benefits in the short term, but in the long term, the sustainable blue carbon value of seagrass ecosystems will outweigh the costs (i.e., there will be positive net post-restoration benefits). As a result of climate change, the study indicates that significant financial investment will be required to restore seagrass.

### Centroid shift of seagrass ecosystems under climate change

**Figure 2:**
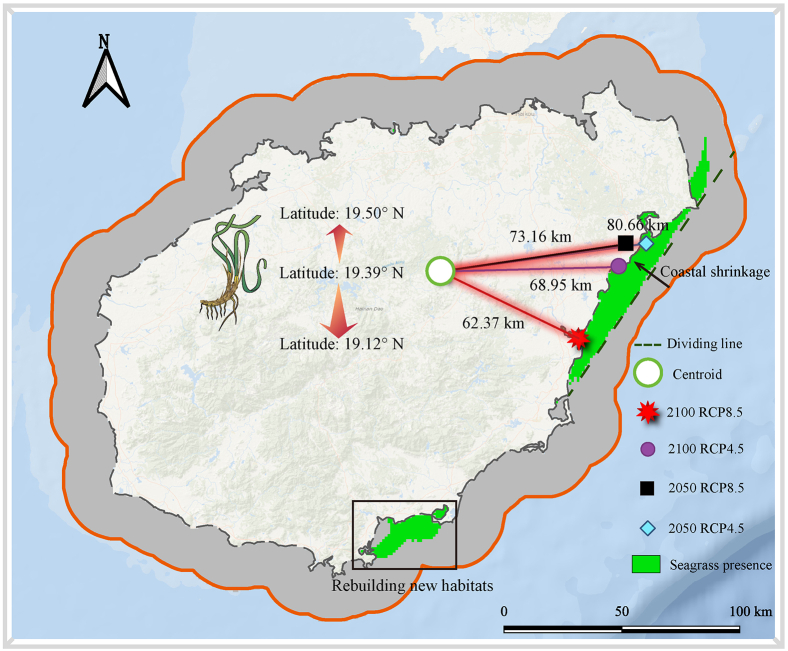
Centroidal shift in seagrass habitat suitability under contrasting scenarios (RCP4.5 and RCP8.5) by 2050 and 2100 The background map uses the seagrass habitat distribution under the RCP8.5 scenario in 2100 to highlight the habitats reestablished by seagrasses under extreme climatic conditions depicted within the black box.

We investigated the centroid shift of the geographic distribution of seagrass species’ suitable habitats under climate change (Figure 2). Our results suggest that under the three climate scenarios (2050 RCP4.5, 2050 RCP8.5, and 2100 RCP4.5), future tropical seagrasses on Hainan Island will shift toward higher latitudes, with the centroid moving from a latitudinal range of 19.39°–19.50°. However, under the RCP8.5 climate scenario in 2100, the centroid of seagrasses will shift toward lower latitudes, i.e., from 19.39° to 19.12°. This intriguing phenomenon could be attributed to the potential development of new climatic refugia along the southern coast of Hainan Island, as a response to anticipated warming conditions in the future. In addition, we found that the magnitude of seagrass migration under both RCP4.5 and RCP8.5 climate scenarios in 2050 is comparatively less pronounced than that anticipated in 2100. Our research indicates that in 2050, despite the loss of 54.8–72% of seagrass coverage, the east coast of Hainan Island will continue to provide a suitable habitat for seagrass growth and there will not be a significant shift in the centroid migration, indicating a relative stability in the core habitat for these seagrass species. However, by 2100 under the RCP8.5 climate scenario, the centroid migrates 62.37 km to the southeast, causing large-scale contraction of seagrass along the coastal direction and rendering most areas of the east coast of Hainan Island unsuitable for seagrass survival.

## Discussion

### Impacts of climate change on seagrass habitats and their ecological implications

How climate determines the distribution of species is a fundamental question in ecology.^36^ The climatic niches of seagrass species are determined by a combination of their functional attributes and interactions with the environmental variables. However, before we can make precise estimates, empirical validation is often required. In the context of global climate change, it becomes imperative to study whether the climatic niche of seagrass species will undergo rapidly shift or remain relatively stable across different geographical regions and time periods (i.e., niche shifts or conservatism).^37^ A comprehensive understanding of these dynamics is vital for seagrasses to adapt to the challenges posed by climate change and maintain their climatic condition in potentially new refugia and uncharted habitats (Figure S3).

To investigate these dynamics, we developed an ensemble model to predict modifications in seagrass habitats and evaluated its performance using different metrics including AUC, omission rate, sensitivity, specificity, and kappa (Table S3). Our research indicates that under future climate change scenarios, unsuitable conditions will rise, curtailing both the spatial extent and dimensions of seagrass habitats. In the most extreme climate change scenario, by 2100, the suitability of seagrass habitats might experience a drastic decline of up to 74.78% (Figure 1). Seagrass species are declining because certain temperature ranges necessary for their thriving are compromised. Coastal areas are experiencing rising water temperatures due to climate change as well. High temperatures can stress seagrasses, preventing them from growing, reproducing, and surviving.^38^ This decline would result in a substantial reduction in distribution of seagrass in the study area. In this scenario, tropical seagrasses on Hainan Island would restrict to thriving solely along the eastern coastline; however, those on the northern and western shores of Hainan Island would no longer thrive, potentially leading to their widespread disappearance or even extinction. Consequently, implementing protective measures is critical to reversing seagrass losses and preventing further harm to seagrass ecosystems. Our results revealed that seagrass habitat loss in Hainan Island align with global patterns observed in other tropical regions. In the Great Barrier Reef, Collier et al.^39^ reported similar vulnerability of seagrass habitats to rising sea temperatures, with projected losses of 40–70% by 2100 under high emission scenarios. Likewise, studies from Southeast Asian seagrass meadows^40^ documented comparable rates of decline due to climate stressors, suggesting a consistent vulnerability pattern of vulnerability across the Indo-Pacific region. However, our projected habitat loss of up to 74.78% by 2100 exceeds estimates from Mediterranean seagrass systems, where Chefaoui et al.^41^ predicted losses of 46.5% for *Cymodocea nodosa* meadows, highlighting the potentially greater vulnerability of tropical seagrass systems to climate change compared to temperate regions.

The projected loss of seagrass habitats carries profound ecological implications beyond carbon storage. Seagrass meadows function as foundation species that create habitat complexity, supporting diverse communities of associated flora and fauna.^8^ Climate-induced seagrass loss could trigger cascading effects throughout coastal food webs on Hainan Island. The decline in seagrass coverage would significantly impact their role as critical nursery habitats for economically important fish and shellfish species in the South China Sea.^9^ The projected 60–75% habitat loss by 2100 could severely reduce recruitment success for species that depend on seagrass during juvenile stages, potentially leading to population declines in species of commercial and ecological importance. The sediment stabilization function of seagrass would also be compromised, likely leading to increased coastal erosion and reduced water clarity. This could trigger negative feedback loops wherein increased turbidity further stresses remaining seagrass communities, accelerating decline rates. Additionally, the loss of wave attenuation services provided by extensive seagrass meadows may also increase the vulnerability of Hainan’s coastal communities to storm events and sea-level rise, particularly along the northern and western shores where our model projects the most substantial habitat losses.

### Climate change affects seagrass blue carbon storage

Seagrass blue carbon storage on Hainan Island faces a considerable threat from climate change. This study reveals that Hainan’s seagrass ecosystems currently sequester 1.194 Tg of carbon (Table 1), with the vast majority (96.5%) residing in sediments rather than plant biomass. This substantial carbon reservoir is consequently vulnerable to the impacts of climate change. The seagrass meadows of Hainan Island are of particular ecological significance, constituting approximately 64% of China’s total seagrass area. Projections indicate that under the moderate RCP4.5 scenario, these ecosystems could experience a carbon loss of 54.78% (0.63 Tg) by 2050 and 60.11% (0.69 Tg) by 2100. Under the more severe RCP8.5 scenario, these losses are projected to escalate to 72.00% (0.84 Tg) by 2050 and 74.78% (0.83 Tg) by 2100. These anticipated losses mirror the patterns observed in habitat suitability projections, with the northern and western coastlines predicted to undergo the most significant declines. The degradation of seagrass meadows leads to the release of carbon stored in both plant tissues and sediments, potentially initiating a positive feedback loop that could exacerbate climate change effects. Ocean warming and increased acidity adversely affect seagrass growth and distribution, thereby diminishing carbon absorption rates. Furthermore, climate change can precipitate extreme events, such as sea-level rise and intensified storms, which destabilize seagrass meadows and their associated carbon stores. These findings underscore the critical importance of protecting extant seagrass habitats, particularly along the eastern coast where models predict some seagrass resilience, even under severe climate scenarios. The results highlight the urgent necessity for conservation initiatives to safeguard these invaluable blue carbon ecosystems.

### Seagrass restoration urgency, costs, and benefits

Climate change’s effects on seagrass ecosystems have gained worldwide attention.^4^^,^^5^^,^^6^ Our analysis revealed that restoring seagrass loss affected by climate change is an urgent and crucial task, and we evaluated its costs and benefits. The economic implications of seagrass carbon losses extend beyond regional considerations to global climate finance mechanisms. Our valuation approach of $16 per tCO_2_ aligns with conservative global carbon pricing mechanisms, though significantly below the social cost of carbon estimated at $177–805 per tCO_2_.^42^ The projected economic losses of $1.02–1.27 billion by 2100 from a relatively small geographic area highlight the potentially massive global economic implications of seagrass habitat degradation worldwide.

We found that compensating for the losses incurred by climate change through seagrass restoration involves significant expenses, such as extensive planting and conservation efforts requiring various resources like planting facilities, technical support, regulatory frameworks, and personnel.^43^^,^^44^ Despite being economically costly and previously deemed unfeasible, current research indicates that the benefits of seagrass restoration outweigh the expenses in the long term, with a projected investment return time of 71 years.^44^ Our results suggest that the cost of restoring seagrasses lost on Hainan Island due to climate change could range from $1.02 billion to $1.26 billion by 2100. Government agencies and supporting organizations primarily finance seagrass restoration projects, and private organizations and non-governmental organizations seldom allocate funds for this cause, hindering the advancement of such initiatives.^43^ Thus, we propose diversifying financing to broaden the range of seagrass restoration efforts and achieve more ambitious objectives in the future. Furthermore, we suggest promoting extensive seagrass plantations in coastal regions worldwide, especially in the South China Sea, to tackle climate change effectively.

### Seagrass migration and range shifts

Global warming will cause tropical seagrasses to migrate and shift. Compared to temperate seagrasses, tropical seagrasses are more deeply impacted by global warming because they originate from ocean areas with stable environmental temperatures and have poor adaptability to temperature changes.^45^ Our results indicated that under future climate change conditions, tropical seagrasses located on Hainan Island in the South China Sea will move to high-latitude regions as climate change disrupting existing marine ecosystems. High-latitude ocean temperatures are lower and more suitable for seagrass habitat and growth, and the centroid of seagrasses has shifted to high-latitude regions in all three climate scenarios. However, under the climate scenario of RCP8.5 in 2100, we observed an exception to this trend, namely a massive offset of seagrass to lower latitudes. In this scenario, a massive expansion of seagrasses habitats was observed along the south coast of Hainan Island, and there no Loss area was detected. This trend indicated the seagrass migration toward lower latitudes. A similar situation occurred in the 2100 RCP4.5 scenario, but unlike it, no large-scale Gain of seagrasses occurs in this climate scenario and, thus, still showed an overall trend of migration toward higher latitudes. Thus, it concludes that seagrass species will move to high-latitude regions under climate change seems premature. For example, seagrass (*Halodule uninervis*) in the Great Barrier Reef is expected to continue to grow vigorously under future temperature rises, while not all seagrass species have strong heat tolerance.^46^ Therefore, our model predicted that although some heat-tolerant seagrasses may benefit from ocean warming, while collective seagrass population must confront the complexities imposed by extreme climate conditions. To combat the challenges posed by climate change, it is imperative to implement more ambitious measures. These measures should aim to limit the global average temperature increase to well below 2°C compared to pre industrial levels and prioritize the protection and restoration of healthy seagrass meadows worldwide.

### Management implications and recommendations for conservation

Seagrass conservation, strategic restoration, and adaptive management will be essential for maintaining the ecological functionality and carbon storage capacity of seagrass ecosystems in the face of climate change. This research offered valuable insights into the future distribution of seagrass meadows on Hainan Island by analyzing centroid shifts in seagrass habitats under various climate scenarios. The findings suggest that, under most climate scenarios for 2050 and 2100, seagrass habitats will migrate toward higher latitudes. This northward movement of seagrass meadows could be a response to evolving environmental conditions. An intriguing observation emerged from the southern coast of Hainan Island for the year 2100, indicating the development of new climatic refugia as well uncharted area. Consequently, the centroid of seagrass meadows shifts southeast. In light of the pronounced seagrass migration toward lower latitudes in 2100, considering long-term projections becomes crucial. Understanding potential shifts in seagrass habitats is vital for devising effective conservation and management strategies. Projected range dynamics can guide the identification of suitable areas for seagrass restoration and the prioritization of conservation efforts. Moreover, the migration of seagrass meadows toward lower latitudes may pose threats to coastal ecosystems and their associated biodiversity.

Based on our habitat suitability projections, we recommend prioritizing specific areas for protection and restoration efforts. The eastern coast of Hainan Island requires immediate conservation attention because it consistently maintains suitable conditions for seagrass under all climate scenarios. These locations act as potential climate refugia. We recommend designating them as marine protected areas with restrictions on coastal development, anchoring, and damaging fishing practices. We also suggest focusing restoration efforts on moderately degraded meadows along the northeastern coast, where conditions are projected to remain favorable through 2100. Additionally, experimental restoration plots could be established along the southern coast. Our models project this area may become suitable by 2100, particularly under the RCP8.5 scenario. This anticipatory restoration strategy would establish founder populations in areas likely to become suitable as climate change progresses. Finally, we advise against major restoration investments along the northwestern coast, as declining suitability under all scenarios suggests limited long-term viability for seagrass ecosystems there.

The anticipated decrease in carbon storage and shift in seagrass habitats underscore the urgent need for proactive conservation and restoration of seagrass. These ecosystems, renowned for nutrient cycling, biodiversity support, and climate regulation, hold considerable ecological and economic significance. While restoring seagrass habitats may pose short-term economic challenges, it is essential to account for their long-term ecological and climate benefits when assessing restoration initiatives. Moreover, seagrass restoration can play a pivotal role in the protection and rejuvenation of coastal ecosystems.

### Conclusion

This study found that Hainan Island’s seagrass ecosystems currently store 1.194 Tg of blue carbon. However, they face significant future degradation from climate change, potentially losing up to 74.78% of their habitat by 2100 under the RCP8.5 scenario. This degradation could release up to 0.83 Tg of stored carbon and lead to economic losses ranging from $305 million by 2050 to $1.27 billion by 2100. Our cost-benefit analysis shows that despite high restoration costs; the long-term climate and ecological benefits greatly outweigh the investment. Our analysis of habitat centroid migration revealed a complex spatial response to climate change. While migration is consistently northward under moderate scenarios, it shifts southward counterintuitively under the most extreme scenario (RCP8.5, 2100). This southward shift occurs because suitable habitats are projected to emerge along Hainan’s southern coast. This finding challenges the assumption of uniform poleward migration and highlights the importance of local conditions in shaping future habitat suitability. We recommend prioritizing the protection of eastern Hainan seagrass habitats, which exhibit resilience under all climate scenarios. We also suggest designating southern coastal areas as potential climate refugia and integrating seagrass conservation into broader climate policies. This research offers spatially specific guidance for conservation planning and improves our understanding of how blue carbon ecosystems contribute to climate change mitigation.

### Limitations of the study

While this research provides valuable insights, it relies on climate models and scenarios that simplify complex environmental interactions. For example, local factors like pollution or sudden weather events, which could further stress seagrass, were not fully explored. Economic valuations of carbon loss and restoration benefits also depend on current market prices, which may shift over time. Additionally, the study focuses on Hainan Island, so applying these results to other regions requires caution, as seagrass species and local conditions vary. Future work could expand data collection to improve predictions and test restoration strategies in emerging habitats.

## Resource availability

### Lead contact

Requests for further information and resources should be directed to and will be fulfilled by the lead contact, Wei Mao (maowei@hainanu.edu.cn).

### Materials availability

This study did not generate new unique reagents.

### Data and code availability

All data reported in this article are included in the article. Additional information will be shared by the lead contact upon request.
No novel code was used in this study.

## Acknowledgments

This study was supported by the Hainan Province Science and Technology Special Fund (ZDYF2023SHFZ172), 10.13039/501100001809National Natural Science Foundation of China (42276235), and the start-up funding from 10.13039/501100005693Hainan University (kyqd20035).

## Author contributions

L.S.: Formal analysis, writing – original draft. B.H.: Conceptualization, formal analysis, methodology, validation, writing – original draft. S.A.: Formal analysis, supervision, writing – review and editing. W.M.: Data curation, funding acquisition, supervision, writing – review and editing. A.C.: Supervision, writing – review and editing.

## Declaration of interests

The authors declare no competing interests.

## STAR★Methods

### Key resources table

**Table:** 

REAGENT or RESOURCE	SOURCE	IDENTIFIER
**Deposited data**		

Seagrass presence data	Global Biodiversity Information Facility	https://www.gbif.org/
Marine environmental data	Bio-ORACLE	https://www.bio-oracle.org/
Carbon stock	Jiang et al.^21^	https://www.sciencedirect.com/science/article/pii/S0025326X17306616
Seagrass restoration costs	Bayraktarov et al.^43^	https://esajournals.onlinelibrary.wiley.com/doi/full/10.1890/15-1077
Ecosystem service values	De Groot et al.^47^	https://www.sciencedirect.com/science/article/pii/S2212041612000101

**Software and algorithms**		

R v4.2.2	R Foundation	https://www.r-project.org/
Python v3.8.8	Python Software Foundation	https://www.python.org/
ArcGIS v10.8	ESRI	https://www.esri.com/
SDMtoolbox v2.6	Brown et al.^48^	https://peerj.com/articles/4095/

### Experimental model and study participant details

This study investigated natural seagrass ecosystems rather than traditional experimental models, focusing on seven seagrass species (*Halophila ovalis*, *Halophila minor*, *Thalassia hemprichii*, *Halodule uninervis*, *Halodule pinifolia*, *Enhalus acoroides*, and *Halophila beccarii*) in their natural marine habitats around Hainan Island, South China Sea (18°09′–20°10′N, 108°37′–111°03′E). Field surveys were conducted during March and August 2021 in coastal waters less than 100 m deep, observing seagrass populations during low tide conditions when colonies were visible, with no experimental manipulation performed. Primary data consisted of GPS-recorded occurrence points (longitude and latitude coordinates) using ICEGPS 610 (NavCom Technology, Inc.), supplemented by occurrence data from published scientific literature (*n* = 28 sources) and the Global Biodiversity Information Facility database (*n* = 39 records). The study area experiences a tropical monsoon climate with annual mean temperatures of 25°C–29°C, providing suitable conditions for tropical seagrass growth. All field surveys were conducted in accordance with local regulations for marine ecological research, with no collection of protected species or destructive sampling performed.

### Method details

#### Database construction

The study area encompasses all coastal regions of Hainan Island, located in the South China Sea, featuring a coastline extending over of 1,619 kilometers and shallow waters of less than 100 meters in deep. Hainan Island is located within a tropical monsoon climate zone, with an annual mean temperature of 25°C–29°C, making it a suitable habitat for seagrass species (Figure S4). The field survey was conducted during March and August 2021 to determine the actual distribution of seagrass on Hainan Island. During low tide, when seagrass colonies were more visible, we used GPS (ICEGPS 610, NavCom Technology, Inc.) to collect occurrence points (longitude and latitude coordinates) of seagrass. Simultaneously, we compiled data on the distribution of seven different types of seagrass (*Halophila ovalis*, *Halophila minor*, *Thalassia hemprichii*, *Halodule uninervis*, *Halodule pinifolia*, *Enhalus acoroides*, and *Halophila beccarii*) from different sources, e.g., scientific research (*n* = 28), including articles, specimen records, and reports,^21^^,^^23^^,^^24^^,^^49^^,^^50^^,^^51^ and online databases (*n* = 39), including the Global Biodiversity Information Facility (https://www.gbif.org/).

We used “dismo” R package^52^ to eliminate duplicate distribution data within a 1 km^2^ grid unit to minimize the potential bias caused by clustered occurrences. The study area was divided into 42 different grid cells as data input based on seagrass distribution data only. To enhance the dependability and precision of the model, we employed the “PresenceAbsence” R package^53^ to randomly generate 10,000 pseudo-absence data points within unit in the study area.

#### Environmental data

The study employed scenario data generated by the Coupled Model Intercomparison Project Phase 5 (CMIP5).^54^ The model chose two representative concentration pathways (RCPs), RCP4.5 and RCP8.5, to project future environmental conditions in 2050 and 2100. Bio-ORACLE 2.2 (https://bio-oracle.org/) ocean data were used to construct the model’s environmental variables,^55^^,^^56^ as shown in Table S4. Five ocean-atmosphere circulation models and CMIP5 provide projections of future environmental data (refer to Table S4). The selected models were used to predict four CMIP5 scenarios (RCP4.5 and RCP8.5 by 2050 and 2100) in the future. The RCP4.5 and RCP8.5 pathways were selected as they provide distinct greenhouse gas concentration trajectories that enabled analysis across a reasonable range of potential futures, including both a stabilization scenario (RCP4.5) as well as a high-emission scenario (RCP8.5) relevant to policy decisions. The data layer’s resolution was 5 arc minutes, approximately 9.2 kilometers on the equator, and we performed kriging interpolation of environmental variables using “gstat” and “raster” R packages^57^^,^^58^ to account the spatial autocorrelation. The data layer’s resolution was later interpolated to 1 km. In ArcGIS, we created a 20-km buffer zone suitable for seagrass distribution, which is considered to be the area where seagrasses may exist,^23^ and all environmental variable data layers were trimmed to fit within the buffer zone.

We used five modeling techniques in “SDM” R package: Generalized Linear Model (GLM),^59^ Generalized Additive Model (GAM),^60^ Multivariate Adaptive Regression Spline (MARS),^61^ Maximum Entropy (MaxEnt),^62^ and Support Vector Machine (SVM).^63^ All techniques were applied to presence data and to the pseudo-absences for the models. We fitted these models into an ensemble model using the “sdm” package^64^ to project range changes in seagrass under future climate change scenarios. At the same time, we assessed the variance between the five models and evaluated them based on several performance metrics, including the Pearson correlation coefficient matrix (Figure S1). For more information on these five algorithms, please refer to their original descriptions.^59^^,^^60^^,^^61^^,^^62^^,^^63^ In our previous study,^23^ we provided present seagrass habitat suitability map data (Figure S2) for the South China Sea Hainan Island, which we compared and analyzed with future seagrass habitat suitability. For the ensemble model, we randomly selected 70% of the data for training and used the remaining 30% for testing. We performed 10-fold cross-validation on each model with 100 repetitions to test the model’s generalization ability, reduce overfitting, and improve model performance. We only included models with three performance metrics (area under curve (AUC), sensitivity, and specificity) greater than or equal to 0.75 (Table S1). We used the “SDMTools” R package^65^ to determine the optimal threshold based on habitat suitability probability and converted habitat suitability maps into binary existence/non-existence maps.

#### Seagrass carbon stock

A dataset of seagrass carbon stocks (Tg C) was compiled based on published studies, data obtained from: Jiang et al.,^21^ Gao et al.,^66^ Jiang et al.^67^ and Meng et al.^68^ In constructing this dataset, we aimed to assess the seagrass ecosystems’ capacity to store blue carbon on Hainan Island.

We divided the seagrass blue carbon stocks into two components: plant carbon stocks and soil carbon stocks. The compiled data divided the soil layer into five depth bands: 0–20, 20–40, 40–60, 60–80 and 80–100 cm (Table S2). We used the “Raster Calculator” in ArcMap 10.8 to convert the binary map of seagrass presence/absence into a seagrass carbon storage data layer, which was used for simulating the change in blue carbon storage of seagrass ecosystems under future climate change.

#### Economic data

The loss of seagrass blue carbon holds substantial economic implications for the future development of Hainan and the broader environment. The valuation of seagrass blue carbon ecosystems was based on the initial blue carbon eco-products issued by the Hainan International Carbon Emission Trading Center (HICETC) under the Internationally Certified Carbon Emission Reduction (ICER) Standard. These tradable carbon credits were assessed at a standardized trading price of USD 16 per tonne of carbon dioxide, according to the exchange rate of May 2022.^69^ We use the blue ecosystem restoration cost data from Bayraktarov et al*.*^43^ To determine the monetary benefits provided by seagrass ecosystems, we used the ecosystem services value database (ESVD), provided by the economics of ecosystems and biodiversity (TEEB) foundation.^47^ We extracted the data on seagrass (*n* = 58) from it and estimated a median restoration cost of seagrass of 74594 US$ ha^−1^ year^−1^ and a median monetary benefit provided after restoring seagrass ecosystems of 1912 US$ ha^−1^ year^−1^. Because the data were collected at different time scales, the units of ecosystem value used in this study were uniformly converted to 2010 US$ ha^−1^ year^−1^.

#### Centroid migration

The study employed the Python-based “SDMtoolbox”^70^ to calculate the centroid migration of seagrass from historical to future distributions. The analysis utilized the “Distribution Changes Between Binary SDMs” tool, which sequentially calculated changes in the habitat range of seagrass for four future climate scenarios, resulting in Gain, Stable, and Loss. Moreover, we used the “Centroid Change (Lines)” tool to calculate trends in the geometric center point migration (i.e., centroid migration) of seagrass suitable habitat under four future climate scenarios.

### Quantification and statistical analysis

Quantification and statistical analysis used in this study including the species distribution modeling, carbon storage, and economic analysis can be found in the method details.
