# Supplementary material for: Seagrass ecosystems in peril: Climate change threatens blue carbon storage and ecosystem services
Source: iScience. 2025 Jun 16;28(7):112909. doi: 10.1016/j.isci.2025.112909 (PMC12268851; doi:10.1016/j.isci.2025.112909)
Supplement: Document S1. Figures S1–S4 and Tables S1–S4 [file mmc1.pdf]

iScience, Volume *28*

Supplemental information

Seagrass ecosystems in peril: Climate change threatens blue carbon storage and ecosystem services

Linlin Song, Bohao He, Shahid Ahmad, Qian Li, Anping Chen, and Wei Mao

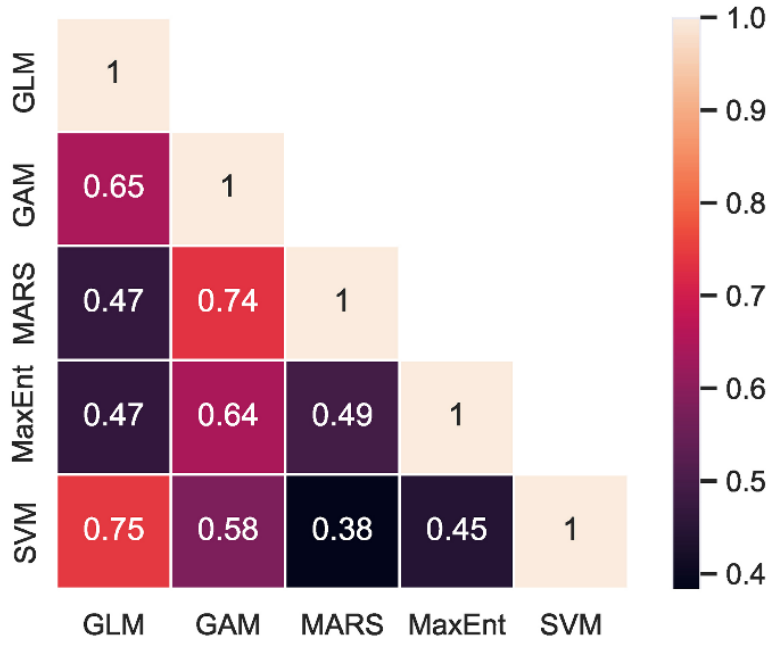

Figure S1. Correlation of five machine learning models in an ensemble model.

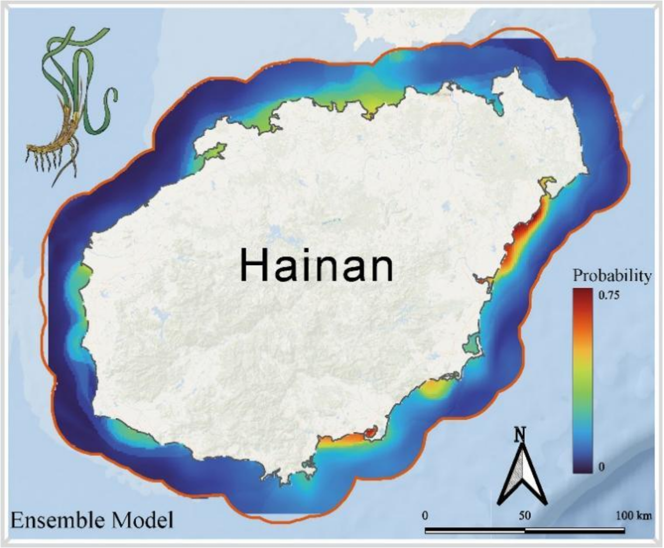

Figure S2. Seagrass suitability habitat distribution.

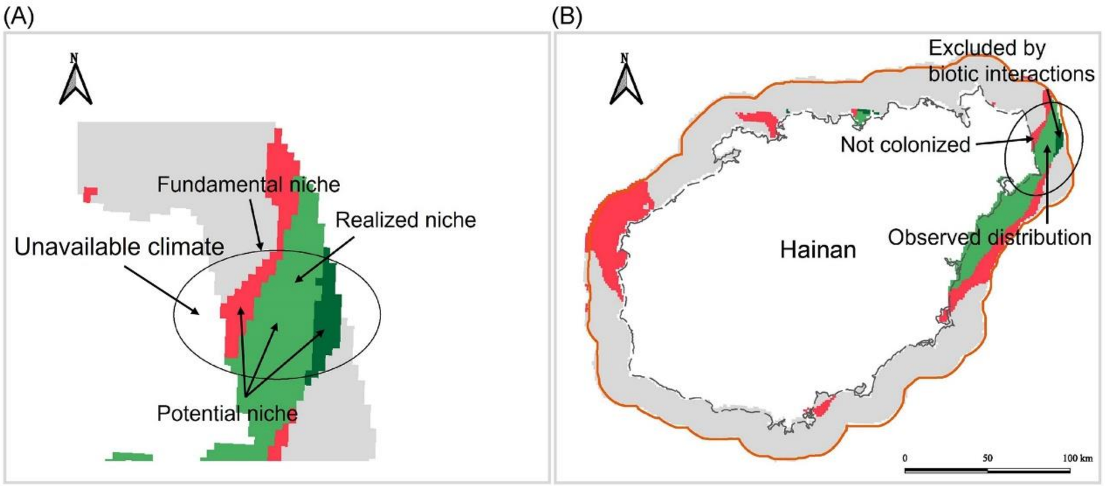

Figure S3. Climate ecological niche changes of seagrasses on Hainan Island, South China Sea.

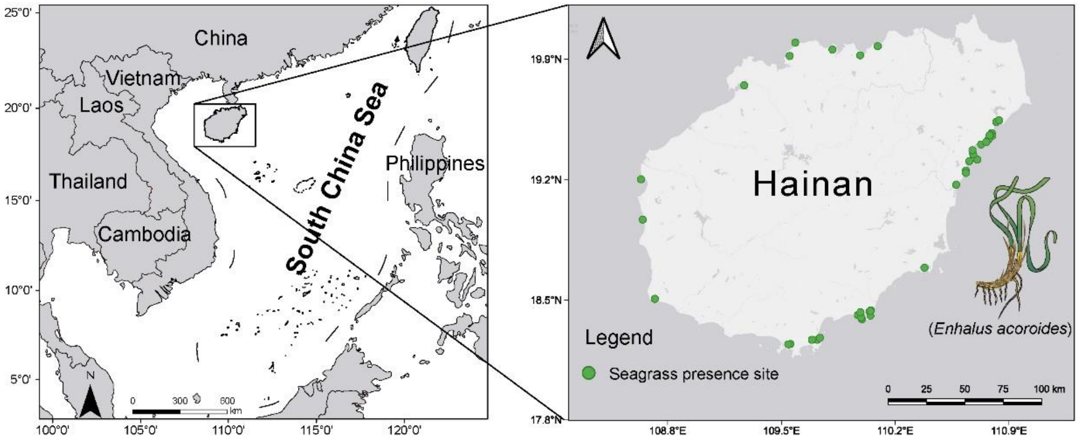

Figure S4. Seagrass distribution sites on Hainan Island, South China Sea. The seagrass distribution site includes seven species of seagrasses, such as *Enhalus acoroides* in Figure.

Table S1. The number of models retained in the modelling of the four climate scenarios.

Scenario	Model	Retention number
	GLM	10
	GAM	9
2050 RCP45	MARS	8
	MaxEnt	8
	SVM	9

2050 RCP85	GLM	3
	GAM	10
	MARS	9
	MaxEnt	9
	SVM	9
2100 RCP45	GLM	10
	GAM	9
	MARS	10
	MaxEnt	10
	SVM	9
2100 RCP85	GLM	7
	GAM	10
	MARS	10
	MaxEnt	9
	SVM	10

Table S2. Depth, bulk density, TOC and soil C stocks of ecosystems in seagrass bed ecosystems in Hainan Island. Values given as mean ± SE.

Depth (cm)	Bulk density (g cm^{-3})	TOC (%)	Soil C stock (MgC ha^{-1})
0-20	1.37±0.20	2.32±0.57Aa	63.57±8.47Aa
20-40	1.47±0.24	2.11±0.37Aa	62.03±4.51Aa
40-60	1.47±0.22	1.82±0.25ABa	53.51±8.25Aa
60-80	1.55±0.09	1.81±0.11Aab	56.11±9.36Aa
80-100	1.59±0.12	1.64±0.42Ab	52.15±6.99Aa
Total			287.37±20.62

Table S3. Predicted validation scores for seagrass suitability habitat using ensemble models under four climate scenarios.

Climate scenario	AUC	Omission (%)	Sensitivity	Specificity	Kappa

2050 RCP45	0.851	0.180	0.735	0.843	0.422
2050 RCP85	0.851	0.182	0.734	0.845	0.362
2100 RCP45	0.869	0.161	0.740	0.872	0.466
2100 RCP85	0.879	0.154	0.741	0.874	0.431

Table S4. Environmental variables used in this study. Five general circulation models (GCM) are descriptions of the CMIP5 used to predict the future distribution of seagrasses.

Notation	Description	Units
Temperature	Mean ocean surface temperature	ºC
Salinity	Mean ocean salinity	PSS
Velocity	Mean currents velocity	m-1
GCM	Original resolution (degrees)	Institution/Country
HadGEM2-ES	1.25, 0.837	UK Met Office Hadley Centre, UK
MPI-ESM-MR	1.875, 1.875	Max Planck Institute for Meteorology, Germany
IPSL-CM5A-LR	3.75, 1.875	IPSL-CM5A-LR Institut Pierre-Simon Laplace, France
NASA/GISS	2.5, 2	Goddard Institute for Space Studies, USA
CNRM-CM5	1.4, 1.4	Météo-France/CNRM, France
